# Supplementary material for: Diverse mutational landscapes in human lymphocytes
Source: Nature. 2022 Aug 10;608(7924):724–32. doi: 10.1038/s41586-022-05072-7 (PMC9402440; doi:10.1038/s41586-022-05072-7)
Supplement: Supplementary file 1 — This file contains Supplementary Figures 1–5; Supplementary Methods; Supplementary Note and Supplementary References [file 41586_2022_5072_MOESM1_ESM.pdf]

nature portfolio

https://doi.org/10.1038/s41586-022-05072-7

Supplementary information

Diverse mutational landscapes in human lymphocytes

In the format provided by the authors and unedited

Supplementary Information

Diverse mutational landscapes in human lymphocytes

Authors: Heather E Machado[1], Emily Mitchell[1,2]†, Nina F Øbro[2,3,4]†, Kirsten Kübler[5-7]†, Megan Davies[2,3,8], Daniel Leongamornlert[1], Alyssa Cull[11], Francesco Maura[9], Mathijs A. Sanders[1,10], Alex TJ Cagan[1], Craig McDonald[2,3,11], Miriam Belmonte[2,3,11], Mairi S. Shepherd[2,3], Felipe A Vieira Braga[1], Robert J Osborne[1,12], Krishnaa Mahbubani[3,13,14], Iñigo Martincorena[1], Elisa Laurenti[2,3], Anthony R Green[2,3], Gad Getz[5-7,15], Paz Polak[16], Kourosh Saeb-Parsy[13,14], Daniel J Hodson[2,3], David Kent[2,3,11]*, Peter J Campbell[1,2]*

Table of Contents

Supplementary Figure 1. 3
Supplementary Methods 4
Section 1. Lymphocyte culture protocol 4
Section 2. Class-switch recombination calling 10
Section 3. Cell culture bias analysis 10
Supplementary Note: Representativeness of lymphocyte colonies 12
References 17

Supplementary Figure 1.

Flow cytometry sorting strategy. (A) Colour density scatterplots of cell surface markers for lymphocytes. One lymphocyte FACs experiment was performed for each of the 11 tissue samples, with the exception of AX001, for which 3 FACs experiments were performed (**Table S1**). Sorting gates are marked with black polygons, labelled with percentages of cells in each gate. (B) Colour density scatterplots for haematopoietic stem and progenitor cells. One HSPC FACs experiment was performed for each of 9 tissue samples (excludes tonsil samples). Details of the antibody panel used for each FACs experiment are listed in **Table S11**.

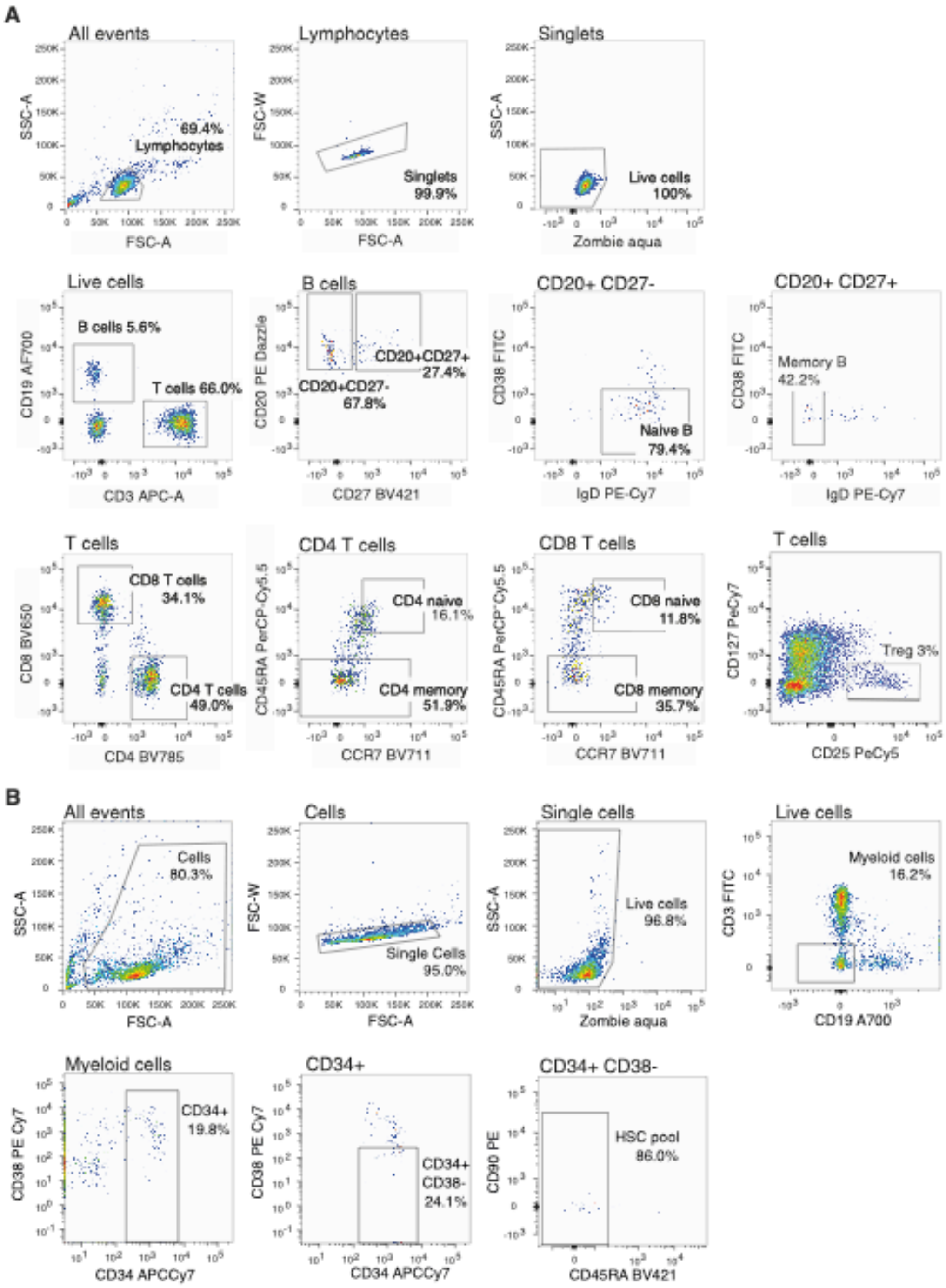

Supplementary Methods

1. Lymphocyte culture protocol
2. Class-switch recombination calling
3. Cell culture bias analysis

Section 1. Lymphocyte culture protocol

Detailed protocol for *in vitro* liquid culture expansion of singly sorted lymphocytes

In this study we developed novel protocols to enable the expansion of single human B and T lymphocytes from single cells into colonies of at least 30 cells. This is a complete protocol starting from frozen viable mononuclear cells (MNCs) and continuing through to DNA extraction of the single cell derived lymphocyte colonies. The extracted DNA can then be used for sequencing library construction.

Materials

Biological materials

- This approach was successfully used on viably frozen human blood mononuclear cells (MNCs) obtained from bone marrow, spleen, tonsil and peripheral blood from seven individuals.
- Any experiments involving human tissues must have ethics approval in accordance with local governmental regulations. Informed consent must also be obtained prior to using samples.

Reagents

Fluorescence activated cell sorting

- Fetal bovine serum (Thermo Fisher Scientific, cat. no. 10270-106)
- Phosphate buffered saline (Sigma-Aldrich, cat. no. D8537-500ML)
- APC anti-human CD3, clone Hit3a (Tonbo Biosciences, cat. no. 20-0039-T100)
- Brilliant violet 785 anti-human CD4, clone OKT4 (BioLegend, cat. no. 317442)
- BV650 mouse anti-human CD8, clone RPA-T8 (BD Biosciences, cat. no. 563821)
- Alexa Fluor 700 anti-human CD19, clone HIB19 (BioLegend, cat. no. 302226)
- PE/Dazzle 594 anti-human CD20, clone 2H7 (BioLegend, cat. no. 302348)
- BV421 mouse anti-human CD27, clone M-T271 (BD Biosciences, cat. no. 562513)
- FITC anti-human CD38, clone HIT2 (BioLegend, cat. no. 303504)
- PerCP/Cyanine5.5 anti-human CD45RA, clone HI100 (BioLegend, cat. no. 304122)
- BV711 anti-human CD197 (CCR7), clone G043H7 (BioLegend, cat. no. 353228)
- PE/Cyanine7 anti-human IgD, clone IA6-2 (BioLegend, cat. no. 348210)
- Zombie Aqua Fixable Viability Kit (BioLegend, cat. no. 423102)
- BD Comp Beads, anti-mouse Ig, κ (BD Biosciences, cat. no. 51-90-9001229)
- BD Comp Beads, negative control (BD Biosciences, cat. no. 51-90-9001291)

Cell culture

- Advanced RPMI 1640 Medium (Sigma-Aldrich, cat. no. R0883-500ML)
- ImmunoCult-XF T Cell Expansion Medium (Stem Cell Technologies, cat. no. 10981)
- Fetal bovine serum (Thermo Fisher Scientific, cat. no. 10270-106)
- Penicillin/streptomycin (Sigma-Aldrich, cat. no. P4333-100ML)
- L-glutamine (Sigma-Aldrich, cat. no. G7513-100ML)

- Anti-IgM (Stratech Scientific Limited, cat. no. 109-006-129-JIR-1mg)
- Recombinant human IL-2 (PeproTech, cat. no. 200-02-500)
- Recombinant human IL-4 (PeproTech, cat. no. 200-04-50)
- Recombinant human IL-15 (PeproTech, cat. no. 200-15-50)
- Recombinant human IL-21 (PeproTech, cat. no. 200-21-100)
- CD40L-HA, recombinant human CD40 ligand (R&D Systems, cat. no. 6420-CL-025)
- HA tag antibody (R&D Systems, cat. no. MAB060)
- ImmunoCult Human CD3/CD28 T cell activator (Stem Cell Technologies, cat. no. 10971)

Sample digest

- Arcturus PicoPure DNA extraction kit (Thermo Fisher Scientific, cat. no. KIT0103)

Equipment

- Multi-purpose refrigerated centrifuge (Eppendorf, model no. 5810 R)
- FACSAria III or FACSAria Fusion (BD Biosciences) with a 100µm nozzle
- 50µm CellTrics cell strainers (Sysmex, cat. no. 04-0042-2313)
- 5mL polypropylene round bottom tubes (Corning, cat. no. 352063)
- 5mL polystyrene round bottom tubes (Corning, cat. no. 352054)
- Sterile 96 well clear flat bottom tissue culture-treated polystyrene cell culture microplates with lids (Corning, cat. no. 353072)
- Twin.tec PCR plate 96, skirted, colorless (Eppendorf, cat. no. 951020401)
- Flat 8 cap strips (Thermo Fisher Scientific, cat. no. AB-0783)
- Standard laboratory equipment including different sized tubes, filter tips, a cell culture incubator, freezer and refrigerator for storing samples and reagents
- CellCelector (ALS Jena)

Procedure

Reagent setup

To reconstitute the human cytokines

1) Prepare all cytokine stocks in PBS 0.1% bovine serum albumin (BSA) as follows:
   a) Recombinant human IL-2
      i) Add 5mL PBS 0.1% BSA to 500µg of lyophilized cytokine
   b) Recombinant human IL-4
      i) Add 500µL PBS 0.1% BSA to 50µg of lyophilized cytokine
   c) Recombinant human IL-21
      i) Add 1mL PBS 0.1% BSA to 100µg of lyophilized cytokine
   d) Recombinant human IL-15
      i) Add 5mL PBS 0.1% BSA to 50µg of lyophilized cytokine
2) Aliquot and store at -80°C. Aliquots can be used for up to one week after thawing.

To reconstitute HA tag antibody

1) Add 200µL PBS to lyophilized antibody, aliquot and store at -20°C. Thawed aliquots are stable in refrigerator.

To prepare the Arcturus PicoPure protease buffer

1) Add 130µL of Arcturus PicoPure DNA reconstitution buffer to each tube of Proteinase K (immediately prior to extraction).
2) Use 17µL of this solution per sample.

Sample preparation

To prepare samples for FACS:

1) Warm 40mL of thawing medium (phosphate buffered saline (PBS) 20% fetal bovine serum (FBS)). Loosen frozen cells in water bath, then pour into warmed thawing medium. In some cases, a wash of the original tube may be desired to increase cell numbers and an additional 1mL of PBS 20% FBS can be used for this.
2) Pellet cells at 500 x g for 5 minutes, remove supernatant and resuspend pellets in 1mL PBS 2% FBS.
3) Reserve cells for unstained controls ($5x10^5$ cells per tube, see below for details on how to prepare these samples) and keep all tubes on ice.
4) Split remaining cell suspension so that there are 3×10^6 cells per tube in a 200µL final volume in PBS 2% FBS and keep these tubes on ice.
5) To this 200µL cell suspension, add the appropriate amount of antibody per tube:

Antibody	Company	Clone	Isotype	Flurophore	Dilution
CD3	Tonbo Biosciences	Hit3a	IgG2a, kappa	APC	1:80
CD4	Biolegend	OKT4	Mouse IgG2b, κ	BV785	1:80
CD8	BD	RPA-T8	Mouse IgG1, κ	BV650	1:40
CD19	Biolegend	HIB19	Mouse IgG1, κ	AF700	1:80
CD20	Biolegend	2H7	Mouse IgG2b, κ	PE Dazzle	1:80
CD27	BD	M-T271	Mouse BALB/c IgG1, κ	BV421	1:80
CD38	Biolegend	HIT2	Mouse IgG1, κ	FITC	1:80
CD45RA	Biolegend	HI100	Mouse IgG2b, κ	PerCP Cy5.5	1:80
CCR7	Biolegend	G043H7	Mouse IgG2a, κ	BV711	1:80
IgD	Biolegend	IA6-2	Mouse IgG2a, κ	PeCy7	1:100
live	Biolegend	n/a	n/a	Zombie aqua	1:400

6) Incubate cells with antibodies for 30 minutes on ice in the dark. Add 1mL PBS 2% FBS and spin cells at 500 x g for 5 minutes.
7) Remove supernatant and resuspend pellets in 500µL PBS 2% FBS. Filter all samples through 50µm cell strainers into fresh FACS tubes and keep on ice.

To prepare single stain controls:

1) Prepare one single stained control per antibody in the panel above by pipetting 100µL PBS 2% FBS into individual polypropylene FACS tubes. Vortex BD Comp beads (both the anti-mouse and negative control bottles). Add one drop of BD Comp Beads, anti-mouse Ig, κ (blue lid) and one drop of BD negative control beads (white lid) into each tube.
2) Add 1µL of the appropriate antibody to each tube and incubate at room temperature for 5 minutes.
3) Add 300µL of PBS 2% FBS to each single stain control tube and place all samples on ice.

To prepare a Zombie Aqua only compensation control:

1) To approximately 2×10^5 cells in a polypropylene tube, add 200µL PBS 2% FBS and add 0.1ul Zombie Aqua,
2) Incubate tube at room temperature for 20 minutes or in the refrigerator for 30 minutes. Following this incubation, pellet cells by centrifuging at 500 x g for 5 minutes.
3) Add 1.5 mL PBS 2% FBS to wash cells and centrifuge sample at 500 x g for 5 minutes. Repeat this wash step once more with 1mL PBS 2% FBS.
4) Resuspend the cell pellet in 300µL PBS 2% FBS and place on ice.

Sorting of B and T cell populations

To sort lymphoid cell populations:

1) Prior to cell sorting, prepare the appropriate expansion medium bases as described below in the B cell- and T cell-specific protocols. Place 96-well flat bottom plates that cells will be sorted into on ice while not in the cell sorting machine.
2) Use the unstained control sample to set voltages for each channel on the FACS machine and use the Zombie Aqua only control to set the live/dead gate. The single stain controls are then used to set up the compensation matrix for each of the detection channels being used. Apply these compensation settings to each of the sample tubes.
   a) Please note that since compensation beads do not allow for the detection of background staining or autofluorescence, it is advisable to run a small amount of the fully stained sample after the initial machine setup.
3) Load the fully stained sample into the flow sorter and set the gates as shown in Supplementary Figure S1.
4) Use the single-cell deposition unit to deposit 1 cell into each of the wells of the 96-well plates, with each well already containing 25-50µL media as described below.
5) If sorting into medium base only (without supplements), following cell sorting add 25µL complete medium plus 2X concentration of each supplement (as indicated below).

Below, we have listed the individual protocols for each cell type:

B cell expansion protocol

Follow these steps to expand single sorted naive and memory B cells:

1) Prepare B cell expansion medium base

Component	Final
Advanced RPMI 1640 Medium	-
FBS	10%
penicillin/streptomycin	1%
L-glutamine	1%

2) Immediately before FACS, add the following supplements to this base: 5µg/ml anti-IgM, 100ng/ml IL-2, 20ng/ml IL-4, 50ng/ml IL-21, 2.5ng/ml CD40L-HA and 1.25µg/ml HA Tag.
3) Add 50µL of this complete B cell expansion medium into the wells of 96-well flat bottom plates. Alternatively, where cell yields are unknown, cells can be sorted into 25µL medium base which can later be topped up to 50µL with 25µL complete B cell medium plus 2X concentration of each supplement.
4) Sort single cells into individual wells of each 96-well plate ensuring that the index sort function has been selected for the FACS machine being used.
5) Maintain cells in a humidified environment for the remainder of the culture period (37°C, 5% CO_2).
6) 1-2 days post-sort, add 25µL of complete B cell expansion medium to each well and repeat every 3-4 days after.
7) 8-12 days after sorting, colonies were harvested either manually or using a CellCelector as detailed below.

T cell expansion protocol

Follow these steps to expand single sorted naive or memory T cells:

1) Prepare T cell expansion medium base

Component	Final

ImmunoCult-XF T Cell Expansion Medium	-
FBS	5%
penicillin/streptomycin	0.5%

2) Immediately before FACS, add the following supplements this base: ImmunoCult CD3/CD28, 100ng/ml IL-2 and 5ng/ml IL-15.
3) Add 50µL of this complete T cell expansion medium into the wells of 96-well flat bottom plates. Where cell yields are unknown, cells can be sorted into 25µL medium base which can later be topped up to 50µL with 25µL complete T cell medium plus 2X concentration of each supplement.
4) 1-2 days post-sort, add 25µL of complete T cell expansion medium to each well and repeat this every 3-4 days after.
5) 10-14 days after sorting, harvest colonies either manually or robotically using a CellCelector as detailed below.

Manual colony picking

For wells that are being picked manually, follow these steps:

1) Pipette up and down in wells to break up colonies and transfer all liquid into a skirted 96-well PCR plate.
2) Place strip tube caps over wells to seal and then pellet cells at 900 x g for 5 minutes.
3) Carefully and in one motion aspirate off liquid from each well, leaving behind a maximum of 3µL of liquid.
4) Add 17uL of proteinase K solution to each well (see above in 'Reagent setup' section for how to prepare this solution) and proceed with cell lysis and digestion steps below.

Colony picking into small volumes (CellCelector protocol)

The automated colony picking function of the CellCelector is used to ensure that (1) the maximum number of cells are collected for small colonies containing <100 cells, and (2) only a fraction of cells (~2000 cells maximum) from large colonies are processed. To pick colonies, follow these steps:

1) Use the CellCelector robot settings (single cell tool with sensor) and after initialising the device, select a safety margin of 1mm, define the pickup diameter of the installed capillary and select the correct plate type.
2) For each experiment, use manual selection alongside live image collection with the camera control set to auto. Using the "activate stage navigation" button, move to the desired well and manually focus using the joystick/control box. To select cell(s), align the crosshairs to the middle of the picking area and click "add single particle".
3) For picking, select "pick particles from list" and ensure that the deck tray matches correctly for the target and deposit plates.
4) Following this, choose "Calibrate pickup position" and proceed through the checklist. Click the "Prepare the tool" button where the "move arm" option allows the arm to bring the capillary into focus and centre on the crosshairs.
5) Slowly move the capillary until the plate base is touched, then withdraw ~0.05 mm. For U-bottom wells, the calibration position should not be far away from the middle of the well. Example "before" and "after" images are given below in **Supplementary Figure 2**.
6) These colonies are picked into the wells of skirted 96-well PCR plates where each well contains 17uL of proteinase K solution (see above in 'Reagent setup' section for how to prepare this solution). Once the colonies have been picked, proceed with cell lysis and digestion steps below.

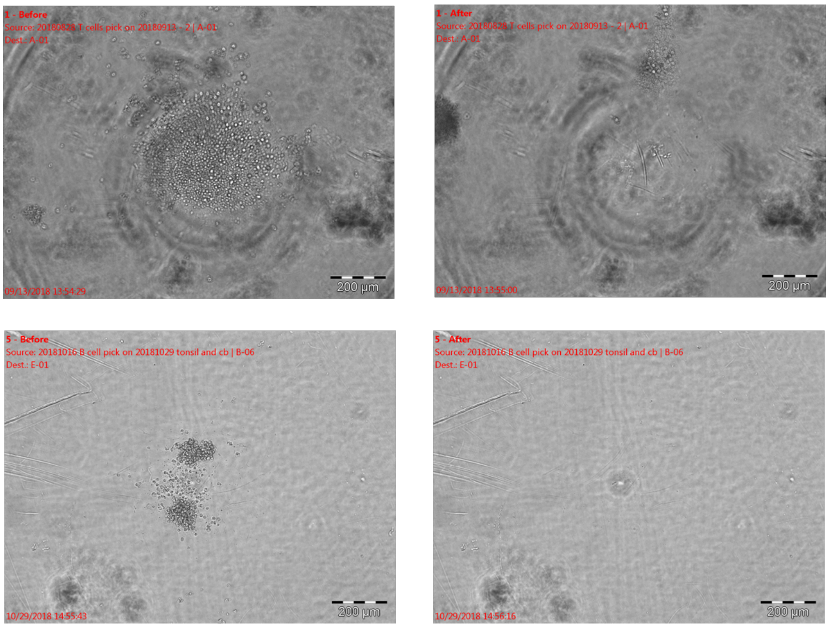

Supplementary Figure 2. CellCelector before and after images. Example images of CD4 naive T cell colony (top left) and naive B cell colony (bottom left) are shown. Following automated colony picking, the majority of each colony was removed (top right and bottom right).

Cell lysis and digestion

Once colonies have been picked into Arcturus PicoPure Proteinase K buffer solution, follow these steps:

1) Ensure that the strip tube caps are securely sealing each well and place the plate on a thermocycler.
2) Run the following program with the lid heated to 110°C:

Step	Temperature (°C)	Time (hours)
1	65	6
2	75	0.5
3	4	hold

3) Plates can then be temporarily stored at -20°C before proceeding with sequencing protocols.

Anticipated Results

We have demonstrated that our protocol reliably generates whole genome sequencing libraries from primary human B and T cell colonies maintained *ex vivo*. The expected number of successfully grown colonies (cloning efficiency) per cell type is indicated below.

**Cell Type**	**Days in Culture**	**Population**	**FACS Strategy**	**Cloning Efficiency (proportion of sorted cells picked for sequencing)**
B Cells	8-12	Naive B Cells	$CD3^{-}CD19^{+}CD20^{+}CD27^{-}CD38^{-}IgD^{+}$	0.022 (range: 0.007-0.129)
		Memory B Cells	$CD3^{-}CD19^{+}CD20^{+}CD27^{+}CD38^{-}IgD^{-}$	0.021 (range: 0.002-0.042)
T Cells	10-14	Naive CD4 T Cells	$CD3^{+}CD4^{+}CCR7^{+}CD45RA^{high}$	0.138 (range: 0.014-0.373)
		Memory CD4 T Cells	$CD3^{+}CD4^{+}CD45RA^{-}$	0.046 (range: 0-0.097)
		Naive CD8 T Cells	$CD3^{+}CD8^{+}CCR7^{+}CD45RA^{high}$	0.047 (range: 0.005-0.210)
		Memory CD8 T Cells	$CD3^{+}CD8^{+}CD45RA^{-}$	0.005 (range: 0-0.018)
		Regulatory T Cells	$CD3^{+}CD4^{+}CD25^{high}CD127^{-}$	0.026 (single experiment)

Section 2. Class-switch recombination calling

CSR events were called through a three-step process:
1) IgCaller CSR calls (default parameters)
2) A joint CSR caller based on read pair count and IGHG3 depth
3) Manual curation

The joint CSR caller consisted of two parts:
1. Read pair count
    a. We used the same aberrant read pairs as selected by BRASS by using the *.brm.bam intermediate file.
    b. Read pairs were selected which spanned CSR target regions as defined by Hübschmann *et al.*[1] and used in IgCaller[2].
    c. These were filtered to keep only IGHM and another CSR target region spanning read pairs
    d. Read pairs were counted for each IGHM CSR event combination
2. IGHG3 normalised coverage
    a. Using mosdepth (Pedersen *et al.* 2018) mean coverage of non-duplicated primary alignments were obtained for two regions;
        i. 14:105991800-105997800 TMEM121 (local control region)
        ii. 14:106242613-106245626 IGHG3_cn (3' of target region)
    b. IGHG3_cn was normalised by TMEM121

A CSR positive event was defined as read pair count >= 2 and IGHG3_cn coverage <= 0.6.

Samples were manually reviewed if 1) IgCaller and the joint CSR caller disagreed or 2) if no CSR was detected by either method but IGHG3_cn <= 0.6.

Section 3. Cell culture bias analysis

We measured potential biases in which lymphocytes successfully formed colonies by looking for increased relatedness among colony-forming lymphocytes or biases in the cell surface marker expression of colony-forming lymphocytes.

Relatedness among colony-forming lymphocytes
We checked for increased relatedness among colony-forming lymphocytes. As none of the lymphocytes sequenced shared a CDR3 sequence (i.e. were from the same clone), we next looked for a skew in the VAF of variants in the colony WGS data compared with bulk WGS data, leveraging the fact that one individual in our current cohort (AX001) was the same person as that enrolled in our earlier study of HSC dynamics where we performed deep targeted resequencing of bulk B and T cell populations[3]. For this individual, we compared the fraction of lymphocyte colonies reporting a given somatic mutation with the variant allele fraction of that mutation in the bulk resequencing data.

Cell surface marker expression
We evaluated potential sources of bias in colony growth by assessing bias in cell surface marker expression. As single cells were FACS index sorted, each cell sorted had a recorded fluorescence level

for each antibody. This enabled us to compare the cell surface expression of various markers between cells seeding colonies picked for sequencing and sorted cells that did not grow colonies. For each FACS experiment, cell type, and marker we performed t-tests to test for a difference in marker expression between lymphocytes that did and those that did not seed colonies. False-discovery rate q-values were calculated using the p-values of all t-tests performed.

Tree sampling

We were able to assess bias in colony growth efficiency associated with phylogenetic lineage by comparing colony whole genome sequencing with bulk sequencing variant allele fractions (VAFs). For this comparison we used the published HSPC phylogenetic tree of donor AX001 and the bulk B and T cell targeted sequencing from Lee-Six et al. 2018. For each cell subset (B or T cells), we identified the set of high confidence variants with at least two alternate reads in the bulk data and calculated the bulk VAF as the number of alternate reads over number of total reads. We calculated the colony WGS VAF for these same sets of variants per cell subset using the per colony genotype calls.

We simulated the process of sampling and growing single-cell derived colonies using a phylogenetic approach, generating genotypes per cell by sampling down the tree with probabilities proportional to the VAFs of variants assigned to each node of the tree, and initially assigning each cell a 5% chance of growth and sequencing (based on observed culture efficiencies). The tree sampling is a progressive process, where a sampling step occurs at each branching of the tree until there are no more remaining nodes with variants. Each step proceeds as follows. Each daughter node containing at least one variant is assigned a probability of being sampled equal to the highest VAF of variants associated with that node. If the sum of all the daughter node VAFs is less than 1, a dummy node is created with the remaining probability to sum to 1. Conversely, if the sum of all the daughter node VAFs is greater than 1, then each VAF is divided by the sum such that the total probability is equal to 1. The daughter nodes are then sampled once based on these probabilities. The genotype of the cell then acquires the main variant of the sampled node, and additional variants of that node are sampled based on their proportional VAF to main variant VAF. Once the tree has been sampled down to the most terminal variant, the final genotype is calculated by sampling each variant of the genotype obtained from tree sampling with a probability equal to the variant calling sensitivity per single-cell derived colony in the main analysis. We then determine if this genotype survives to sequencing probabilistically, based on its final culture efficiency (see below). Sampling of cells continues until the same number of cells per subset are successfully “sequenced” as in the main analysis (67 naive/memory B cells and 171 naive/memory T cells for AX001). This completes one full simulation, and the genotypes are used to calculate the set of VAFs for that sample.

We assigned a culture efficiency to each cell produced through the tree sampling based on a range of phylogenetic lineage biases, from 0 to 60%. We set the baseline culture efficiency to 5%. For each simulation, each node was assigned a particular percent deviation in culture efficiency which was drawn from a uniform distribution of negative to positive X% (where X is the level of bias). The final culture efficiency of a cell is calculated by walking down the lineage of the cell and adding the proportional amount of deviation in culture efficiency with each subsequent node.

Supplementary Note: Representativeness of lymphocyte colonies

The dataset relies on novel protocols for producing single-cell-derived B and T cell colonies. Clearly, the results that emerge are only generalisable if the lymphocyte colonies we have sequenced are representative of the wider pool of lymphocytes within the individual research subjects. To address this question, we have undertaken three analyses: (1) estimating the efficiency of the culture system for each cell type; (2) assessing the flow cytometric measurements of lymphocytes that successfully seeded colonies and those that did not; and (3) comparing clonality of mutations in cultured lymphocytes to bulk populations from the donors.

Efficiency of culture system

The culture efficiencies we achieved ranged from 0.5% to 14%, depending on the cell type. The naive CD4+ T cells had the highest efficiency at 14%, while the lowest efficiency cell type was memory CD8+ T cells, with an average efficiency of 0.5%. As a result, these latter cells are rare in our analysis (a total of 8 cells out of 635 lymphocytes) and represent 8% of the memory T cells sequenced, the remaining 92 being CD4+ memory T cells. The remaining cell types had efficiencies in the range of 2-5% (naive B, 2%; memory B, 2%; memory CD4+ T, 5%; naive CD8+ T, 5%; Tregs, 3%). Culture efficiencies for each of the different FACS experiments, broken down by research subject and cell type, are reported in **Supplementary Table S1**.

Flow cytometric parameters of successful versus unsuccessful colonies

We leveraged the index-sorting data of our single cells to compare the cell surface expression of various markers between cells seeding colonies picked for sequencing and sorted cells that did not successfully grow colonies. Reassuringly, for naive and memory B cells, there were no significant differences between successful and unsuccessful cells in fluorescence intensity of CD19, CD20, CD27 or IgD for any of the flow-sorts in any of the research subjects (**Extended Figure 1**). We did find some significant differences in fluorescence intensity for T cells between those that successfully grew and those that did not. However, these were not consistent across different individuals, nor were they always in the same direction. One difference, seen in two research subjects, was that T cells higher in CCR7 were somewhat more likely to generate successful colonies in culture. For naive T cells, we gated for CCR7^{high}, attempting to exclude terminally differentiated effector T cells that are CD45RA^{high} (like the naive T cells) but CCR7^{low}. It is possible, however, that the CCR7^{high} gate for naive T cells still captured some terminally differentiated effector cells, which may not proliferate in culture. For the memory T cells, we included the entire CCR7 gate, attempting to culture both effector and central memory T cells. The CCR7^{high} central memory T cells grew somewhat better than the CCR7^{low} effector memory cells. Reassuringly, though, we nonetheless successfully cultured representative numbers of CCR7^{low} colonies, as can be seen in the overlaid data points in **Extended Figure 1**.

Taken together, then, the index flow-sorting data suggest that any biases in colony efficiency within sorted populations are negligible.

Clonality of cultured lymphocytes versus bulk populations

Efficiency rates of 2-5% in our culture system mean that we are accessing a sizable subset of the whole lymphocyte population within an individual, given the large total population size of circulating

lymphocytes. Nonetheless, there remains the possibility that the population that does successfully grow in culture is biased in some way – perhaps they preferentially derive from fitter clones or from cells with particular sub-lineage biases, for example.

To address whether there might be clone-to-clone biases in which cells successfully seeded colonies, we studied the phylogenetic relationships among the cells from a given research subject. First, none of the lymphocytes sequenced shared a CDR3 sequence, indicating that we did not sequence multiple colonies derived from the same *in vivo* clonal expansion. Second, we leveraged the fact that one individual in our current cohort (AX001) was the same person as that enrolled in our earlier study of HSC dynamics, where we performed deep targeted resequencing of bulk B and T cell populations[3]. For this individual, we compared the fraction of lymphocyte colonies reporting a given somatic mutation with the variant allele fraction of that mutation in the bulk resequencing data. Reassuringly, there was a strong correlation between the VAF of mutations in colonies versus those in the bulk lymphocyte populations (**Extended Figure 2A**).

The major question raised is whether the distribution of somatic mutations found in the colonies, shown in **Extended Figure 2A**, followed the distribution expected, given the frequency of those mutations in bulk lymphocyte populations. For example, there appears to be a skewing of the residuals, with a wider spread of points below the regression line than above it in the figure. To address whether this skewing represents sampling noise or culture bias, we describe first the phylogenetic / lineage-tracing aspects of the detected mutations and second an analysis using non-parametric bootstrapping to assess whether there is evidence for bias in culture efficiency across lineages.

<u>1. Phylogenetics and lineage relationships of somatic mutations</u>

All somatic cells in an individual can trace their pedigree back through a series of cell divisions to the fertilised egg[4]. Each of those cell divisions can generate new somatic mutations that become permanent lineage marks found in all subsequent descendants from that cell – the earliest branch-points in phylogenetic trees constructed this way represent cell divisions that predate the split of placenta from the main foetus, for example[5]. The total number of lymphocytes in an adult human is in the order of trillions (10^{12}), whereas our deep sequencing for somatic mutations can detect mutations down to a frequency of ~0.003 with confidence. This means that it is only those mutations that occurred during embryonic and foetal development or mutations carried by monoclonal expansions (such as seen in clonal haematopoiesis) that will be detectable with our approach. In the individual studied here, we did not find evidence of sizable adult-onset clonal expansions in either haematopoietic stem cells or lymphocytes[3], so we would expect that the mutations detected through deep sequencing represent variants acquired during development.

In our earlier publication on this individual, we reconstructed the phylogenetic tree from his haematopoietic stem and progenitor cells, and showed that this tree included branches that represented early embryonic cell divisions[3]. We therefore mapped mutations called in the lymphocyte colonies and deep sequencing of B and T cells onto this phylogenetic tree – shown in **Supplementary Figure 3** below.

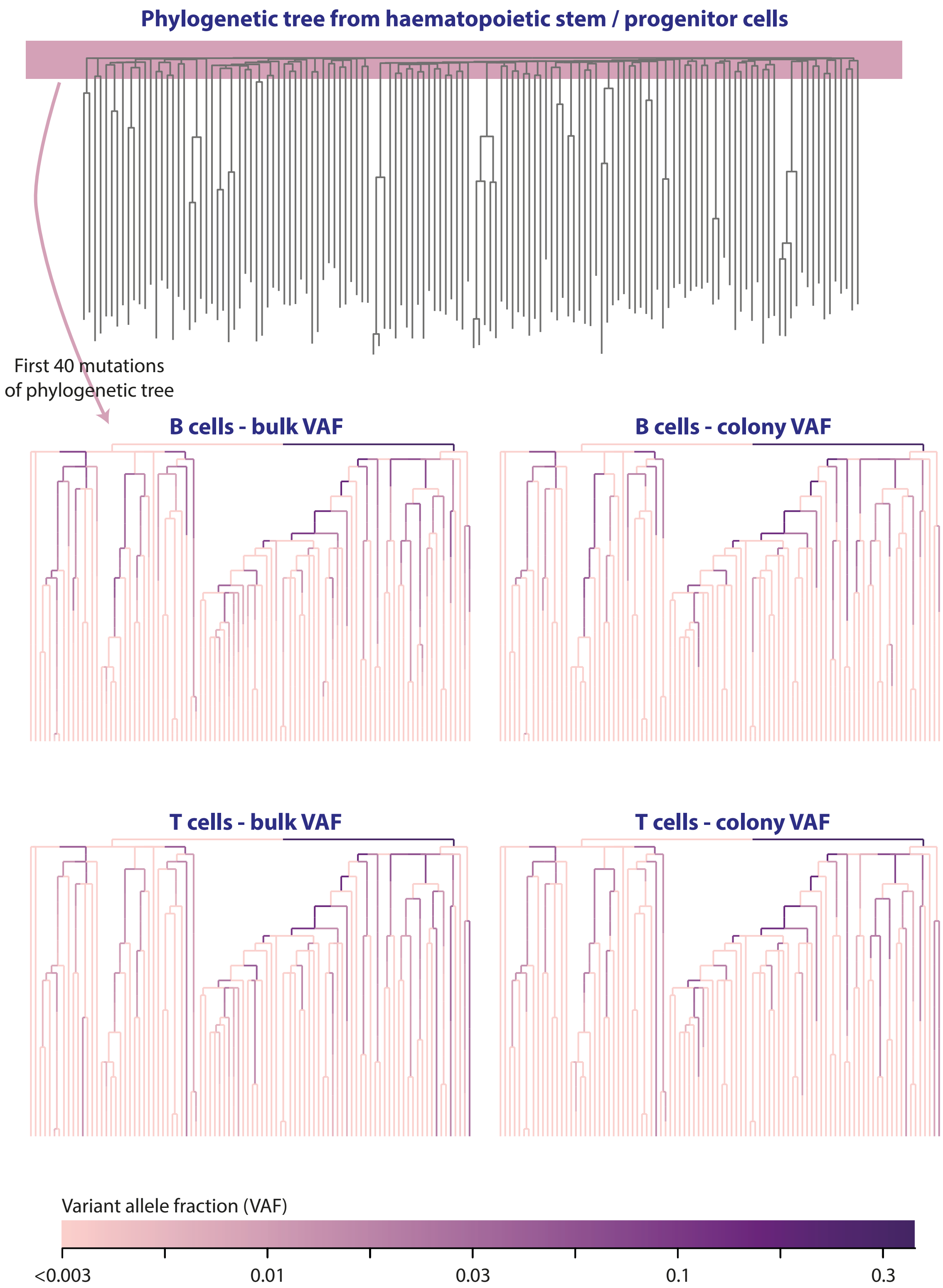

Supplementary Figure 3. Variant allele fraction of mutations acquired during embryonic development in bulk and cultured lymphocyte populations from research subject AX001. A phylogenetic tree was reconstructed for AX001 from whole genome sequencing of haematopoietic stem and progenitor cells, as previously described[3] (top panel). The first 40 mutations of molecular time in this tree represent mutations acquired during foetal development[3]. Deep targeted sequencing was performed for those developmental mutations occurring in sufficiently unique genomic sequence for successful RNA bait design (about 47% of all mutations). The four panels below the overall tree show the same phylogenetic tree as the top panel, but zoomed in on the first 40 mutations of

molecular time, with branches coloured by variant allele fraction from pink (low VAF/not targeted) to purple (high VAF) of those mutations in bulk DNA (left panels) or colonies (right panels) from B (upper) or T (lower) lymphocytes.

Several reassuring observations emerge from this analysis. First, the lymphocyte colonies draw broadly from across the foetal development lineage tree – the lymphocytes that we successfully cultured are truly polyclonal, deriving from both daughter cells of some of the earliest cell divisions in the embryo. Second, the fractional contributions of different developmental lineages to the lymphocyte colonies broadly match those observed in bulk lymphocytes – the same lineages that contribute the highest fraction of cells to the bulk lymphocyte populations in the adult subject contribute similar fractions to the colonies (purple bars in **Supplementary Figure 3**). Third, comparing lineage VAFs between B and T lymphocytes reveals broadly similar distributions – these mutations occurred before the developmental split between B and T lymphocytes, and it is therefore reassuring that mutations are represented in approximately equal proportions across the two cell types.

2. Bootstrapping to quantify the extent of culture bias

As can be seen in **Extended Figure 2A**, the VAFs of mutations observed in bulk DNA versus in lymphocyte colonies showed spread around the expected equality line, with more points below that line than above – this could indicate systematic bias in culture efficiency among different lymphocyte lineages or, alternatively, could be entirely consistent with the expected effects of Poisson sampling. In statistical terms, the question is whether the *residuals* (that is, the deviation of points from the y=x equality line) follow the distribution expected just from sampling, or whether their distribution is more consistent with additional effects from factors such as culture bias.

Standard statistical methods for assessing distributions of residuals (such as the Shapiro-Wilk test for normality) do not work in our scenario for the following two reasons:

- The counts of colonies reporting a mutation follow a Poisson distribution rather than a normal distribution for a given mean – this leads to skewed residuals, especially when the expected VAF (μ) from bulk DNA is low (the skewness of a Poisson distribution is $1/\sqrt{\mu}$);
- The mutations are not independent of one another – this means that the VAF of mutations from the same developmental lineage correlate with one another.

Therefore, to address the extent of any potential bias in the observed VAFs for colonies versus bulk DNA, we used nonparametric bootstrapping. Essentially, we draw samples of lymphocytes from the bulk lymphocyte trees shown in **Supplementary Figure 3**. Each lineage is sampled at the frequency dictated by its VAF in the bulk lymphocyte population (including unobserved lineages, since these are evident as branch-points in the tree where the VAFs of descendant branches do not sum to the VAF of in the inbound branch). For any given lineage, we can introduce a simulated random bias into its culture efficiency by evolving this trait over the tree – for example, if the average culture efficiency is 0.05 then a culture bias of 20% would equate to efficiencies drawn from the range 0.04 to 0.06. For each bootstrap iteration, we sample simulated 'lymphocytes' from across the tree, maintaining those that are simulated to culture successfully using these lineage-specific 'efficiencies', until we acquire the correct number of 'colonies'. Each iteration then generates a bootstrapped sample of colony VAFs to compare against the bulk lymphocyte VAFs. We generated 10,000 bootstrap iterations in this way for every level of culture bias from 0 to 0.9 in increments of 0.05.

We used QQ plots to assess our observed residuals against the expected distribution of residuals estimated from the bootstrap iterations. Where the level of culture efficiency bias is underestimated, the observed residual quantiles will be above the equality line in the QQ plots (because the residuals

are systematically larger than expected); where culture bias is overestimated, the residual quantiles will fall below the equality line. The expected ranges of residuals and the QQ plots are shown in **Supplementary Figure 4**.

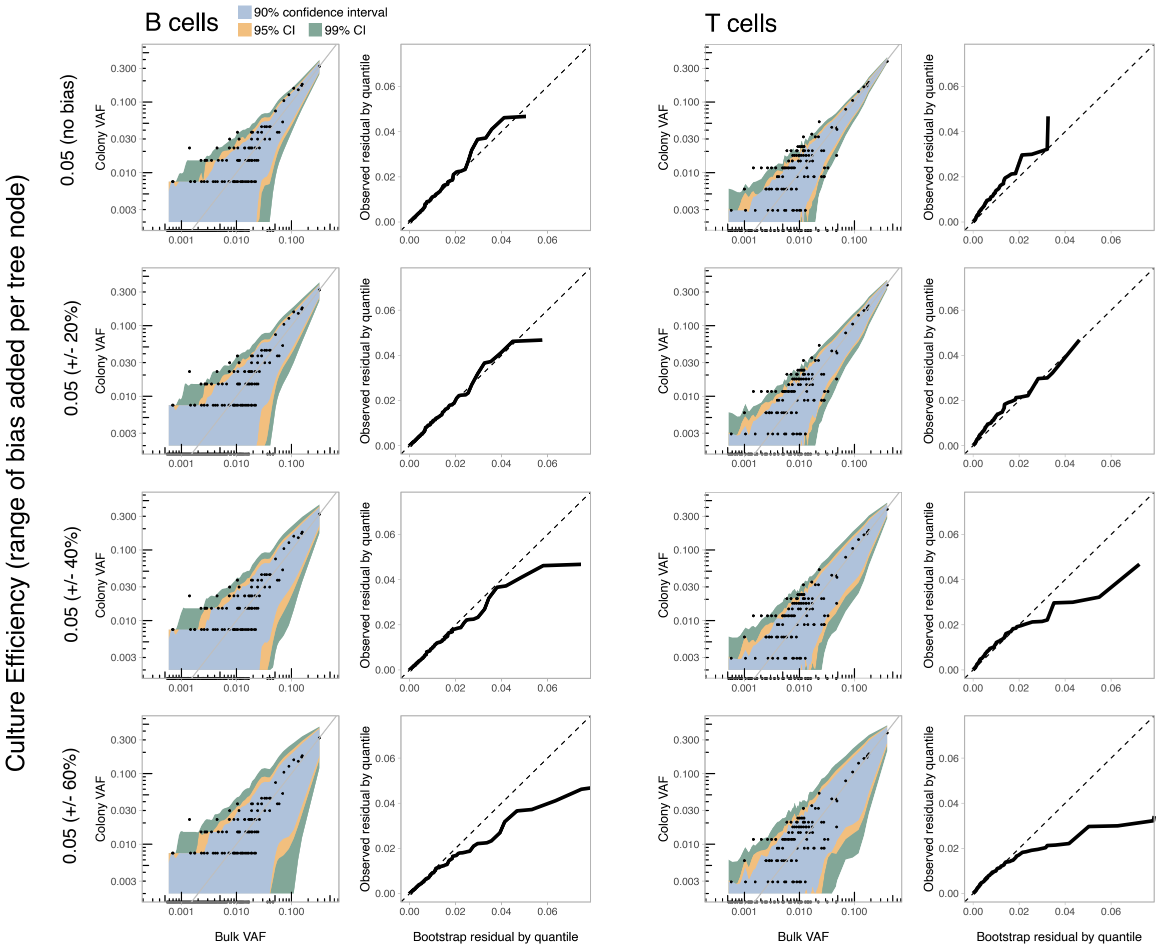

Supplementary Figure 4. Distributions of observed versus expected residuals for colony VAFs under different levels of bias in culture efficiency. The four rows represent different levels of assumed culture efficiency in the nonparametric bootstrap samples (0%, 20%, 40% and 60%), with bootstraps for B lymphocytes on the left and T lymphocytes on the right. Within each condition, the left graph shows the observed data for VAFs in bulk DNA (x axis) versus colonies (y axis) as black points distributed around the x=y line in grey. The shaded areas represent the estimated 90% (blue), 95% (orange) and 99% (green) intervals for the distributions of residuals from the bootstrap samples. The right graph shows the QQ plot with the expected (bootstrap) quantiles on the x axis and the observed quantiles on the y axis. The x=y equality line is shown as a dashed black line.

Reassuringly, we see a strong correlation between the bulk and colony VAFs – the QQ plots for the condition of zero culture bias are close to the equality line (top row of plots in **Supplementary Figure 4**) indicating that the observed residuals are distributed similar to expectation. Nonetheless, for both B and T cells, there is a small systematic deviation above the equality line at zero culture bias; as the level of bias in culture efficiency increases, the QQ plots initially move closer to the equality line and then beyond. If we express this using an 'area under the curve' (AUC; specifically the area above or below the equality line), we see that this area is closest to 0 when the bias in culture efficiency is ~20% for B and T cells (**Supplementary Figure 5**). Thus, if the discrepancy between colony and bulk VAFs were entirely explained by lineage-specific bias in culture efficiency, we estimate that it would equate

to a bias of ~20% for both B and T cells (culture probabilities typically within the range 0.04 to 0.06 for a given lineage with a standard deviation of 0.01).

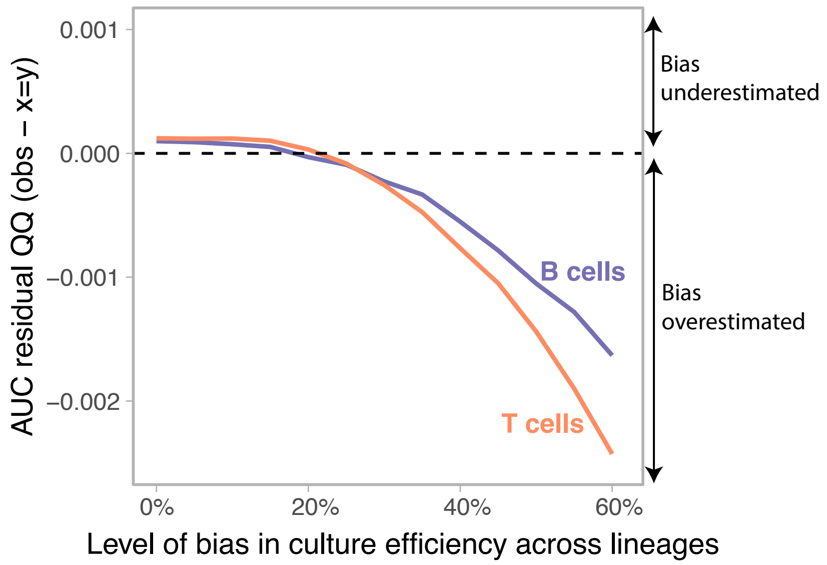

Supplementary Figure 5. Area under the curve (AUC) for QQ plots for bootstraps performed with different levels of bias in culture efficiency. The AUC is measured as the net area above or below the equality line in the QQ plots as shown in Supplementary Figure 2. Positive values for AUC suggest that the bias is systematically underestimated, whereas negative values suggest it is overestimated. The lines cross 0 when the level of bias in culture efficiency is ~20% for both T and B lymphocytes.

References

1. Hübschmann, D. *et al.* Mutational mechanisms shaping the coding and noncoding genome of germinal center derived B-cell lymphomas. *Leukemia* **35**, 2002–2016 (2021).
2. Nadeu, F. *et al.* IgCaller for reconstructing immunoglobulin gene rearrangements and oncogenic translocations from whole-genome sequencing in lymphoid neoplasms. *Nat. Commun.* **11**, 1–11 (2020).
3. Lee-Six, H. *et al.* Population dynamics of normal human blood inferred from somatic mutations. *Nature* **561**, 473–478 (2018).
4. Stratton, M. R., Campbell, P. J. & Futreal, P. A. The cancer genome. *Nature* **458**, 719–724 (2009).
5. Spencer Chapman, M. *et al.* Lineage tracing of human development through somatic mutations. *Nature* **595**, 85–90 (2021).
