# Supplementary material for: Diverse mutational landscapes in human lymphocytes
Source: Nature. 2022 Aug 10;608(7924):724–32. doi: 10.1038/s41586-022-05072-7 (PMC9402440; doi:10.1038/s41586-022-05072-7)
Supplement: Supplementary file 2 — Reporting Summary [file 41586_2022_5072_MOESM2_ESM.pdf]

nature portfolio

Corresponding author(s): Peter Campbell
David Kent

Last updated by author(s): Jun 5, 2022

Reporting Summary

Nature Portfolio wishes to improve the reproducibility of the work that we publish. This form provides structure for consistency and transparency in reporting. For further information on Nature Portfolio policies, see our Editorial Policies and the Editorial Policy Checklist.

Statistics

For all statistical analyses, confirm that the following items are present in the figure legend, table legend, main text, or Methods section.

n/a	Confirmed	
☐	☒	The exact sample size (*n*) for each experimental group/condition, given as a discrete number and unit of measurement
☐	☒	A statement on whether measurements were taken from distinct samples or whether the same sample was measured repeatedly
☐	☒	The statistical test(s) used AND whether they are one- or two-sided<br>*Only common tests should be described solely by name; describe more complex techniques in the Methods section.*
☐	☒	A description of all covariates tested
☐	☒	A description of any assumptions or corrections, such as tests of normality and adjustment for multiple comparisons
☐	☒	A full description of the statistical parameters including central tendency (e.g. means) or other basic estimates (e.g. regression coefficient) AND variation (e.g. standard deviation) or associated estimates of uncertainty (e.g. confidence intervals)
☐	☒	For null hypothesis testing, the test statistic (e.g. *F*, *t*, *r*) with confidence intervals, effect sizes, degrees of freedom and *P* value noted<br>*Give P values as exact values whenever suitable.*
☒	☐	For Bayesian analysis, information on the choice of priors and Markov chain Monte Carlo settings
☐	☒	For hierarchical and complex designs, identification of the appropriate level for tests and full reporting of outcomes
☐	☒	Estimates of effect sizes (e.g. Cohen's *d*, Pearson's *r*), indicating how they were calculated

Our web collection on statistics for biologists contains articles on many of the points above.

Software and code

Policy information about availability of computer code

Data collection	No software was used for data collection.
Data analysis	Open source programs used (also stated in manuscript): BWA v0.7.17, CaVEMan v1.11.2, Pindel v3.1.2, BRASS v6.1.2, ASCAT 4.0.1, R v4.0.2, AnnotateBRASS v3, SigProfiler 1.0.0, IgCaller v1.2, FIMO v5.4.1, MCAST v5.4.1, Telomerecat v4.0.2, FlowJo v10.7.1, FCS Express v7.10.0007, Rstudio v1.4.1073.<br>R packages used: hdp v0.1.5, sigfit v2.2., Rsamtools v2.2.3, MASS v7.3.57, GenomicRanges v1.38.0, plyr v1.8.5, ggplot2 v3.2.1, foreach v1.4.8, doParallel v1.0.16, reshape2 v1.4.3, sigfit v2.2, stringr v1.4.0, dplyr v0.8.4, RColorBrewer v1.1.2, BSgenome.Hsapiens.UCSC.hg19 v1.4.3, selectiveInference v1.2.5, gamsel v1.8.1, mgcv v1.8.31, grid v3.5.1, gridExtra v2.3, ggpubr v0.2.4, tidymv v3.2.0, GenomicFeatures v1.42.1, nrmisc v, tidyverse v1.3.0, magrittr v1.5, rtracklayer v1.50.0, BSgenome.Hsapiens.1000genomes.hs37d5 v0.99.1, cowplot v1.0.0<br><br>Custom code made available (also stated in manuscript): https://github.com/machadoheather/lymphocyte_somatic_mutation<br><br>No commercial software used.

For manuscripts utilizing custom algorithms or software that are central to the research but not yet described in published literature, software must be made available to editors and reviewers. We strongly encourage code deposition in a community repository (e.g. GitHub). See the Nature Portfolio guidelines for submitting code & software for further information.

Data

Policy information about availability of data

All manuscripts must include a data availability statement. This statement should provide the following information, where applicable:

- Accession codes, unique identifiers, or web links for publicly available datasets
- A description of any restrictions on data availability
- For clinical datasets or third party data, please ensure that the statement adheres to our policy

Sequence data that support the findings of this study have been deposited in the European Genome-Phenome Archive (https://www.ebi.ac.uk/ega/home), accession EGAD00001008107. The 149 epigenetic datasets are from the ENCODE and IHEC studies and are described in Table S9. The genomic feature datasets are as follows (and described in Table S4): FeatureID: Data_source
ALU_rep_dist_log10: doi.org/10.1038/s41586-019-1913-9
centromere_dist_log10: doi.org/10.1038/s41586-019-1913-9
cpg_islands_dist_log10: doi.org/10.1038/s41586-019-1913-9
direct_rep_dist_log10: doi.org/10.1038/s41586-019-1913-9
DNA_rep_dist_log10: doi.org/10.1038/s41586-019-1913-9
DNAMethylSBS: doi.org/10.1038/s41586-019-1913-9
g4_dist_log10: doi.org/10.1038/s41586-019-1913-9
gc_content_value: doi.org/10.1038/s41586-019-1913-9
gene_dens_1e6: doi.org/10.1038/s41586-019-1913-9
H2A.Z: doi.org/10.1038/s41586-019-1913-9
H3K27me3: doi.org/10.1038/s41586-019-1913-9
H3K36me3: doi.org/10.1038/s41586-019-1913-9
H3K4me1: doi.org/10.1038/s41586-019-1913-9
H3K4me2: doi.org/10.1038/s41586-019-1913-9
H3K4me3: doi.org/10.1038/s41586-019-1913-9
H3K79me2: doi.org/10.1038/s41586-019-1913-9
H3K9ac: doi.org/10.1038/s41586-019-1913-9
H3K9me3: doi.org/10.1038/s41586-019-1913-9
H4K20me1: doi.org/10.1038/s41586-019-1913-9
L1_rep_dist_log10: doi.org/10.1038/s41586-019-1913-9
L2_rep_dist_log10: doi.org/10.1038/s41586-019-1913-9
LAD_dens_1e6: doi.org/10.1038/s41586-019-1913-9
LTR_rep_dist_log10: doi.org/10.1038/s41586-019-1913-9
MIR_rep_dist_log10: doi.org/10.1038/s41586-019-1913-9
recomb_rate_nearest_value: doi.org/10.1038/s41586-019-1913-9
rep_timing_Gm: http://genome.ucsc.edu/cgi-bin/hgFileUi?db=hg19&g=wgEncodeUwRepliSeq
RNAseq: doi.org/10.1038/s41586-019-1913-9
short_tandem_rep_dens_3e3: doi.org/10.1038/s41586-019-1913-9
SIMPLE_REPEAT_rep_dist_log10: doi.org/10.1038/s41586-019-1913-9
TAD_b_dist_log10: doi.org/10.1038/s41586-019-1913-9
telomere_dist_log10: doi.org/10.1038/s41586-019-1913-9
triplex_mirror_rep_dist_log10: doi.org/10.1038/s41586-019-1913-9

Field-specific reporting

Please select the one below that is the best fit for your research. If you are not sure, read the appropriate sections before making your selection.

☒ Life sciences ☐ Behavioural & social sciences ☐ Ecological, evolutionary & environmental sciences

For a reference copy of the document with all sections, see nature.com/documents/nr-reporting-summary-flat.pdf

Life sciences study design

All studies must disclose on these points even when the disclosure is negative.

Sample size	We optimised the number of individuals (7) and number of genomes per cell subset per individual (average of 102 genomes per individual) to describe the general mutational landscape per cell subset across a range of ages. No power calculation was performed, and there was no target effect size. The samples were chosen to have a broad distribution across ages, from birth (cord blood) up to 81 years of age, where we would expect to start seeing clonal haematopoiesis. Samples were spaced as evenly as possible across ages, with the limitation of pediatric samples, for which only 4 year old samples were obtainable. Previous studies had found an average mutation rate of 16 mutations per cell per year, which indicated that sampling 7 individuals along the described age range would allow for a statistically significant estimates of mutation rates in lymphocytes.
Data exclusions	Per pre-established criteria, genomes with a sequencing depth of less than 6x or and average VAF of less than 20% were excluded. This removed a total 39 genomes: 15 KX001 genomes, 12 KX002 genomes and 12 KX003 genomes.

Replication	While the specific samples used have been exhausted, most of the results from this study should be generally reproducible in separate healthy individuals of the same age, using the protocols and code included in this manuscript.
Randomization	This is not relevant to our study. All individuals were hematopoietically normal, and there was no test versus control groups.
Blinding	Blinding was not relevant to our study. The study only included samples of normal lymphocytes, and no tests were performed that required blinding.

Reporting for specific materials, systems and methods

We require information from authors about some types of materials, experimental systems and methods used in many studies. Here, indicate whether each material, system or method listed is relevant to your study. If you are not sure if a list item applies to your research, read the appropriate section before selecting a response.

Materials & experimental systems

n/a	Involved in the study	
☐	☒	Antibodies
☒	☐	Eukaryotic cell lines
☒	☐	Palaeontology and archaeology
☒	☐	Animals and other organisms
☐	☒	Human research participants
☒	☐	Clinical data
☒	☐	Dual use research of concern

Methods

n/a	Involved in the study	
☒	☐	ChIP-seq
☐	☒	Flow cytometry
☒	☐	MRI-based neuroimaging

Antibodies

Antibodies used

Antibody; Company; Clone; Catalogue Number; Flurophore; Dilution; Panel
CD3; BD; HIT3a; 555339; FITC; 1:500; HSPC_nonAX001
CD90; Biolgend; 5E10; 328110; PE; 1:50; HSPC_nonAX001
CD49f; BD; GoH3; 551129; PECy5; 1:100; HSPC_nonAX001
CD19; Biolgend; HIB19; 302226; A700; 1:300; HSPC_nonAX001
CD34; Biolgend; 581; 343514; APCCy7; 1:100; HSPC_nonAX001
Zombie ; Biolgend; NA; 423101; Aqua; 1:2000; HSPC_nonAX001
CD38; Biolgend; HIT2; 303516; PECy7; 1:100; HSPC_nonAX001
CD45RA; Biolgend; HI100; 304130; BV421; 1:100; HSPC_nonAX001
CD38; BD; HIT2; 560982; FITC; 1:100; HSPC_AX001
CD135; Biolegend; BV10A4H2; 313306; PE; 1:100; HSPC_AX001
CD34; BD; 581; 560710; PE-Cy7; 1:100; HSPC_AX001
CD90; BD; 5E10; 561971; APC; 1:100; HSPC_AX001
CD10; Biolegend; HI10a; 312212; APC-Cy7; 1:100; HSPC_AX001
CD45RA; BD; HI100; 562298; V450; 1:100; HSPC_AX001
CD3; Tonbo Biosciences; Hit3a; 20-0039-T100; APC; 1:80; lymphocyte_nonTreg
CD4; Biolegend; OKT4; 317441; BV785; 1:80; lymphocyte_nonTreg
CD8; BD; RPA-T8; 563821; BV650; 1:40; lymphocyte_nonTreg
CD19; Biolegend; HIB19; 302226; AF700; 1:80; lymphocyte_nonTreg
CD20; Biolegend; 2H7; 302347; PE Dazzle; 1:80; lymphocyte_nonTreg
CD27; BD; M-T271; 562513; BV421; 1:80; lymphocyte_nonTreg
CD38; Biolegend; HIT2; 356610; FITC; 1:80; lymphocyte_nonTreg
CD45RA; Biolegend; HI100; 560362; PerCP Cy5.5; 1:80; lymphocyte_nonTreg
CCR7; Biolegend; G043H7; 353227; BV711; 1:80; lymphocyte_nonTreg
IgD; Biolegend; IA6-2; 348209; PeCy7; 1:100; lymphocyte_nonTreg
live; Biolegend; n/a; 423101; Zombie aqua; 1:400; lymphocyte_nonTreg
CD3; Tonbo Biosciences; Hit3a; 20-0039-T100; APC; 1:80; Treg
CD4; Biolegend; OKT4; 317441; BV785; 1:80; Treg
CD8; BD; RPA-T8; 563821; BV650; 1:40; Treg
CD19; Biolegend; HIB19; 302226; AF700; 1:80; Treg
CD45RA; Biolegend; HI100; 560362; PerCP Cy5.5; 1:80; Treg
CD56; Biolegend; 39D5; 355503; PE; 1:80; Treg
CCR7; Biolegend; G043H7; 353227; FITC; 1:80; Treg
CD25; Biolegend; BC96; 302607; PeCy5; 1:80; Treg
CD127; Biolegend; A019D5; 351319; PeCy7; 1:80; Treg
CD69; Biolegend; FN50; 310921; AF700; 1:80; Treg
CD103; Biolegend; Ber-ACT8; 350213; BV421; 1:80; Treg
CCR9; Biolegend; L053E8; 358903; PE; 1:80; Treg
live; Biolegend; n/a; 423101; Zombie aqua; 1:400; Treg

Validation

These were all previously validated commercially available antibodies. Manufacturer validation and references for each can be found:
Antibody; Flurophore; Company; Catalogue Number; ManufacturerInformation
CCR7; FITC; Biolegend; 353227; https://www.biolegend.com/fr-ch/products/fitc-anti-human-cd197-ccr7-antibody-7537

CCR9; PE; Biolegend; 358903; https://www.biolegend.com/de-de/products/pe-anti-human-cd199-ccr9-antibody-8761
CD10; APC-Cy7; Biolegend; 312212; https://www.biolegend.com/fr-ch/products/apc-cyanine7-anti-human-cd10-antibody-4034
CD103; BV421; Biolegend; 350213; https://www.biolegend.com/de-de/products/brilliant-violet-421-anti-human-cd103-integrin-alphae-antibody-9746
CD127; PeCy7; Biolegend; 351319; https://www.biolegend.com/fr-fr/products/pe-cyanine7-anti-human-cd127-il-7ralpha-antibody-7216
CD135; PE; Biolegend; 313306; https://www.biolegend.com/fr-ch/products/pe-anti-human-cd135-flt-3-flk-2-antibody-2359
CD19; AF700; Biolegend; 302226; https://www.biolegend.com/it-it/products/alexa-fluor-700-anti-human-cd19-antibody-3399
CD20; PE Dazzle; Biolegend; 302347; https://www.biolegend.com/de-de/products/pe-dazzle-594-anti-human-cd20-antibody-10436
CD25; PeCy5; Biolegend; 302607; https://www.biolegend.com/en-gb/products/pe-cyanine5-anti-human-cd25-antibody-617
CD27; BV421; BD; 562513; https://www.bdbiosciences.com/en-us/products/reagents/flow-cytometry-reagents/research-reagents/single-color-antibodies-ruo/bv421-mouse-anti-human-cd27.562513
CD3; FITC; BD; 555339; https://www.bdbiosciences.com/en-us/products/reagents/flow-cytometry-reagents/research-reagents/single-color-antibodies-ruo/fitc-mouse-anti-human-cd3.555339
CD3; APC; Tonbo Biosciences; 20-0039-T100; https://tonbobio.com/products/apc-anti-human-cd3-hit3a
CD34; APCCy7; Biolgend; 343514; https://www.biolegend.com/fr-lu/products/apc-cyanine7-anti-human-cd34-antibody-6159
CD34; PE-Cy7; BD; 560710; https://www.bdbiosciences.com/en-gb/products/reagents/flow-cytometry-reagents/research-reagents/single-color-antibodies-ruo/pe-cy-7-mouse-anti-human-cd34.560710
CD38; PECy7; Biolgend; 303516; https://www.biolegend.com/fr-ch/products/pe-cyanine7-anti-human-cd38-antibody-5418
CD38; FITC; Biolegend; 356610; https://www.biolegend.com/en-ie/products/fitc-anti-human-cd38-antibody-14047
CD38; FITC; BD; 560982; https://www.bdbiosciences.com/en-us/products/reagents/flow-cytometry-reagents/research-reagents/single-color-antibodies-ruo/fitc-mouse-anti-human-cd38.560982
CD4; BV785; Biolegend; 317441; https://www.biolegend.com/fr-lu/products/brilliant-violet-785-anti-human-cd4-antibody-7978
CD45RA; BV421; Biolgend; 304130; https://www.biolegend.com/de-de/products/brilliant-violet-421-anti-human-cd45ra-antibody-7200
CD45RA; PerCP Cy5.5; Biolegend; 560362; https://www.biolegend.com/en-us/search-results/percp-cyanine5-5-anti-human-cd45ra-antibody-4241
CD45RA; V450; BD; 562298; https://www.bdbiosciences.com/en-gb/products/reagents/flow-cytometry-reagents/research-reagents/single-color-antibodies-ruo/v450-mouse-anti-human-cd45ra.560363
CD49f; PECy5; BD; 551129; https://www.bdbiosciences.com/zh-cn/products/reagents/flow-cytometry-reagents/research-reagents/single-color-antibodies-ruo/pe-cy-5-rat-anti-human-cd49f.551129
CD56; PE; Biolegend; 355503; https://www.biolegend.com/en-us/products/pe-anti-human-cd56-subset-msc-marker-antibody-8191
CD69; AF700; Biolegend; 310921; https://www.biolegend.com/fr-ch/products/alexa-fluor-700-anti-human-cd69-antibody-3425
CD8; BV650; BD; 563821; https://www.bdbiosciences.com/ko-kr/products/reagents/flow-cytometry-reagents/research-reagents/single-color-antibodies-ruo/bv650-mouse-anti-human-cd8.563821
CD90; PE; Biolgend; 328110; https://www.biolegend.com/it-it/products/pe-anti-human-cd90-thy1-antibody-4114
CD90; APC; BD; 561971; https://www.bdbiosciences.com/en-ca/products/reagents/flow-cytometry-reagents/research-reagents/single-color-antibodies-ruo/fitc-mouse-anti-human-cd90.555595
IgD; PeCy7; Biolegend; 348209; https://www.biolegend.com/en-us/search-results/pe-cyanine7-anti-human-igd-antibody-6996
live; Zombie aqua; Biolegend; 423101; https://www.biolegend.com/fr-fr/products/zombie-aqua-fixable-viability-kit-8444

Human research participants

Policy information about studies involving human research participants

Population characteristics

Describe the covariate-relevant population characteristics of the human research participants (e.g. age, gender, genotypic information, past and current diagnosis and treatment categories). If you filled out the behavioural & social sciences study design questions and have nothing to add here, write "See above."

Recruitment

Describe how participants were recruited. Outline any potential self-selection bias or other biases that may be present and how these are likely to impact results.

Ethics oversight

Identify the organization(s) that approved the study protocol.

Note that full information on the approval of the study protocol must also be provided in the manuscript.

Flow Cytometry

Plots

Confirm that:

☒ The axis labels state the marker and fluorochrome used (e.g. CD4-FITC).

☒ The axis scales are clearly visible. Include numbers along axes only for bottom left plot of group (a 'group' is an analysis of identical markers).

☒ All plots are contour plots with outliers or pseudocolor plots.

☒ A numerical value for number of cells or percentage (with statistics) is provided.

Methodology

Sample preparation

All samples were received as viably frozen human blood mononuclear cells (MNCs) obtained from bone marrow, spleen, tonsil and peripheral blood from seven individuals.

Instrument	Sorting was performed on FACSAria III or FACSAria Fusion (BD Biosciences).
Software	During the sort data were collected using the FACSAria III or FACSAria Fusion (BD Biosciences) default software. Plotting of the gating strategy supplementary figure was done on FlowJo and plotting of the index sort data for the culture bias supplementary figure was done using FCS Express.
Cell population abundance	This study used no data on relative cell abundances for any of the samples.
Gating strategy	The FSC/SSC gate was set using the manual gating tool to exclude debris and small particles and to identify the lymphocyte fraction of cells as displayed in Supplementary Figure S1. Further gating was as follows: naive B lymphocytes (CD3-CD19+CD20+CD27-CD38-IgD+), memory B lymphocytes (CD3-CD19+CD20+CD27+CD38-IgD-), naive T lymphocytes (CD3+CD4/CD8+CCR7+CD45RAhigh), memory T lymphocytes (CD3+CD4/CD8+CD45RA-), regulatory T cells (Tregs: CD3+CD4+CD25highCD127-) and HSPCs (CD3-CD19-CD34+CD38-CD90+CD45RA-). HSPCs from AX001 included HSCs (CD34+CD38-) and progenitors (CD34+CD38+CD10-/dim).

☒ Tick this box to confirm that a figure exemplifying the gating strategy is provided in the Supplementary Information.

March 2021
